# Remodeling of the Metabolome during Early Frog Development

**DOI:** 10.1371/journal.pone.0016881

**Published:** 2011-02-04

**Authors:** Livia Vastag, Paul Jorgensen, Leonid Peshkin, Ru Wei, Joshua D. Rabinowitz, Marc W. Kirschner

**Affiliations:** 1 Carl Icahn Laboratory, Department of Chemistry and Lewis-Sigler Institute for Integrative Genomics, Princeton University, Princeton, New Jersey, United States of America; 2 Department of Systems Biology, Harvard Medical School, Boston, Massachusetts, United States of America; 3 Broad Institute of MIT and Harvard, Cambridge, Massachusetts, United States of America; University of Birmingham, United Kingdom

## Abstract

A rapid series of synchronous cell divisions initiates embryogenesis in many animal species, including the frog *Xenopus laevis*. After many of these cleavage cycles, the nuclear to cytoplasmic ratio increases sufficiently to somehow cause cell cycles to elongate and become asynchronous at the mid-blastula transition (MBT). We have discovered that an unanticipated remodeling of core metabolic pathways occurs during the cleavage cycles and the MBT in *X.laevis*, as evidenced by widespread changes in metabolite abundance. While many of the changes in metabolite abundance were consistently observed, it was also evident that different female frogs laid eggs with different levels of at least some metabolites. Metabolite tracing with heavy isotopes demonstrated that alanine is consumed to generate energy for the early embryo. dATP pools were found to decline during the MBT and we have confirmed that maternal pools of dNTPs are functionally exhausted at the onset of the MBT. Our results support an alternative hypothesis that the cell cycle lengthening at the MBT is triggered not by a limiting maternal protein, as is usually proposed, but by a decline in dNTP pools brought about by the exponentially increasing demands of DNA synthesis.

## Introduction

The metabolic pathways that route the traffic of small molecules through cells arose very early in evolution and have been strongly conserved. Animals generally rely on the same metabolic network as the earliest single-celled eukaryotes, although in some cases, such as the biosynthesis of many amino acids, entire pathways have become unnecessary and have been lost. But the deep conservation of metabolic pathways does not imply that all animal cells have the same metabolism. Indeed, an animal’s metabolism is maintained by transfer of small molecules between different organs and biochemical differentiation is so prevalent as to be the norm. For instance, it is well appreciated that hepatocytes, myocytes, and neurons have specialized metabolic capabilities and requirements. Recently it has been demonstrated that even mouse embryonic stem cells have a specialized metabolic state [1], [2].

Metabolism is also very different in quiescent versus proliferating cells. There is an increasingly deep understanding of the metabolic attributes of rapidly growing cells, particularly cancerous cells, and how these attributes are programmed by growth factor signaling in mouse and human cells [3]. Continual cell proliferation requires not only that cells duplicate and divide their genetic material but also that they double in size. To grow in size, rapidly proliferating cells require a constant supply of building blocks, such as amino acids, and reducing equivalents, such as NADPH. At least in cultured cells, this anabolic state appears to be sustained primarily by the consumption of glucose and glutamine [4].

The embryos of the African clawed frog *X. laevis* have been of great use to researchers studying the cell cycle, development, and the connections between the two. As in many, if not most, animal species, fertilization of the *X.laevis* egg initiates a series of rapid and synchronous cell division cycles [5]. These cleavage cycles represent the minimal cell cycle, alternating between genome replication (S-phase) and genome segregation (M-phase) and lacking gap phases (G1 and G2-phase) and cell cycle checkpoints. Little to no cell growth occurs during the cleavage cycles. Once embryos reach a threshold ratio of DNA:cytoplasm after 12–13 cleavage cycles [6], [7], [8], the cleavage cycles slow and lose synchrony, an event termed the mid-blastula transition (MBT). For the remainder of the blastula period, cells continue to cycle but the length of the cell cycles increases, in inverse proportion to cell size [8], [9]. Although the MBT is also associated with an increased level of zygotic transcription and the appearance of cell motility, for the purposes of this paper we consider the MBT solely from the perspective of the cell cycle. It is generally thought that some maternal protein stored in the egg, potentially a replication origin factor [10], becomes limiting at the MBT, thereby reporting the DNA:cytoplasm ratio to the cell cycle machinery.

The relationships between metabolism, the cell cycle, and development are unclear and demand further exploration. Mass-spectrometry based techniques now allow numerous metabolites from a single source (e.g. a cell extract) to be rapidly identified and quantitated. The emerging field of metabolomics uses such techniques in attempting to systematically describe the metabolome, the complete set of all metabolites present in a biological sample. Using metabolomic approaches, we have investigated whether metabolism changes during the very earliest stages of frog development. We have discovered that widespread changes occur to metabolite abundance during this period. Following up on some of the most intriguing changes led to insights into energy metabolism in early embryos, as well as support for an alternative model of the cell cycle lengthening at the MBT.

## Results and Discussion

### Widespread changes to metabolite pools during early *X. laevis* development

**Figure 1:**
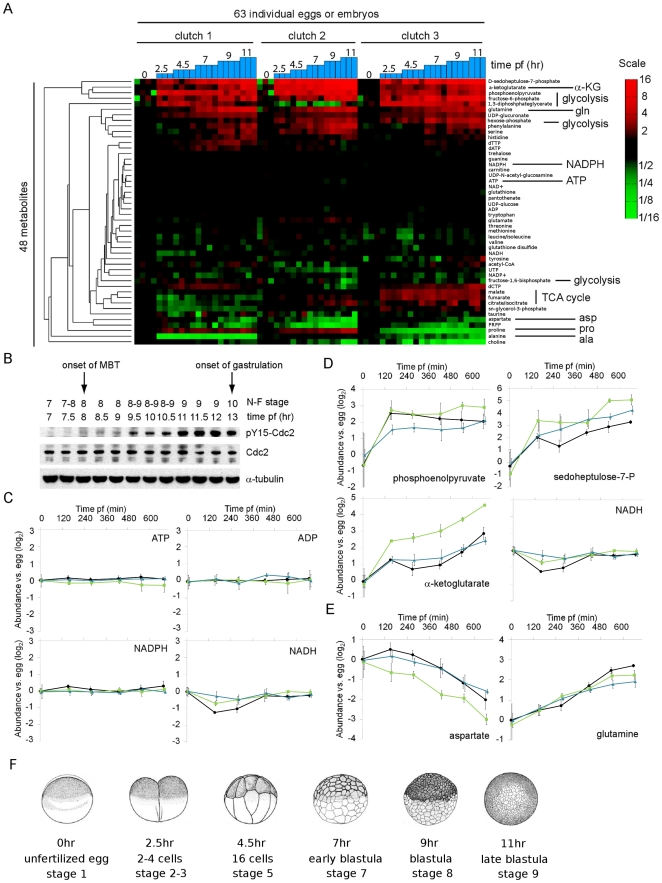
Remodeling of the metabolome during early *X.laevis* development. (**A**) Dendrogram and heat map showing the wide-spread remodeling of the metabolome in early development. On three different days, three different male/female pairs were bred in vitro to obtain clutches of unfertilized eggs (time = 0) and early embryos at various times post-fertilization (pf) at18°C. Single eggs or embryos were rapidly quenched, extracted, and subjected to metabolomic analysis. Metabolite levels were expressed relative to the average value for that metabolite in the egg samples. The ratios were calculated separately for the three different clutches. The metabolites (rows) were then clustered hierarchically and ratios plotted on a color scale. For each time point n = 3–4. (**B**) The timing of key events in early *X.laevis* development at 18°C. Groups of 5 embryos from the same mating were harvested at the indicated time points post-fertilization (pf) and extracts prepared for western blotting. The Nieuwkoop-Faber (N-F) stage of embryos was recorded [48]. Y15 on Cdc2 phosphorylation rose 8 hours pf indicating that cell cycles had begun to slow and embryos were entering the MBT. Bottle cells appeared on embryos 13 hours pf indicating the start of gastrulation. (**C–E**) Plots of individual metabolite abundance relative to the value measured in eggs during early development. For each single egg or embryo, each metabolite abundance was divided by the average egg value for that metabolite. This ratio was then log_2_ transformed (n = 3-4± 1SD). These data are the same data presented in (A). The three clutches were plotted separately (black: clutch 1, green: clutch 2, blue: clutch 3). (**F**) Illustration of the embryos harvested at the indicated hours post fertilization. The corresponding Nieuwkoop-Faber stages are also indicated.

When the abundant yolk platelets are excluded, *X.laevis* eggs have a volume of ∼500 nL, equivalent to ∼250,000 mammalian tissue culture cells. In preliminary experiments, we found that we were able to measure the abundance of numerous metabolites in single *X.laevis* eggs and embryos. (Here-in, once eggs have been fertilized, they are called embryos.) By measuring metabolites in single eggs and embryos, we could avoid the averaging implicit in many biological measurements and determine the extent of variation between individuals. For each measurement, single eggs or embryos were rapidly quenched in a 2∶2∶1 acetonitrile:methanol:water mixture at −20°C. Soluble metabolites were extracted from the insoluble cellular material (e.g. protein, DNA, RNA) and subjected to metabolomic analysis with a liquid chromatography- tandem mass spectrometry (LC-MS/MS) method. This platform has been previously demonstrated to provide reproducible and linear measurements of more than 100 common metabolites [11]. We were able to detect a core set of 48 metabolites in single eggs or embryos in nearly all of our experiments (Figure 1A). We focused our analyses on this robust set of 48 metabolites, which represents a broad cross-section of central metabolic pathways, including amino acids, nucleotides, and phosphorylated sugars.

We prepared extracts from single eggs and from single embryos in the earliest stages of development. Embryos were collected 2.5, 4.5, 7, 9, and 11 hours after in vitro fertilization. The time course was repeated with three crosses of unique male and female frogs. The stages of development examined include the cleavage cycles, the MBT, and the late blastula period (Figure 1F). Phosphorylation of tyrosine-15 (pY15) in Cdc2 increased 8 hr after fertilization, which marks the onset of the MBT (Figure 1B) [12], [13]. The time course ends prior to gastrulation (∼13 hr post-fertilization).

Widespread changes occur to the metabolomes of frog embryos in the time between egg fertilization and the late blastula stage 11 hours later (Figure 1A). The abundance of at least 15 of the core 48 metabolites changed during these early stages of development. To confirm this surprising result and the validity of the single embryo measurements from the LC-MS/MS system in the Rabinowitz laboratory (Lewis-Sigler Institute for Integrative Genomics, Carl Icahn Laboratory, Princeton University, Princeton, NJ, U.S.A), we performed a second set of time courses, altered many of the details of quenching and extraction, and then quantified metabolites with a different LC-MS/MS system in the Mootha laboratory (Broad Institute of MIT and Harvard, Cambridge, MA, USA). Although each laboratory’s system is calibrated to measure a different set of metabolites, the great majority of overlapping metabolites showed consistent patterns following fertilization (Figure S1).

Throughout these early stages of development, the key energetic and reductive currencies ATP, NADPH, and NADH remained nearly constant with little embryo-to-embryo variation in abundance (Figure 1C). But intermediates in the core metabolic pathways that sustain ATP, NADPH, and NADH levels did change in abundance. Most of the components of the glycolytic pathway that we measured showed reproducible increases (Figure 1A, Table S1), with the phosphoenolpyruvate (PEP) pool showing a particularly sharp ∼4–8 fold expansion just following fertilization (Figure 1D). Like the glycolytic intermediates, the sedoheptulose-7-phosphate (S7P) pool expanded greatly in early development (∼8–32 fold, Figure 1D). S7P is the only constituent of the non-oxidative portion of the pentose phosphate pathway (non-ox PPP) that we measured and the non-ox PPP is connected to the glycolytic pathway via reversible reactions. The rise in S7P concentration likely reflects an equilibrium between S7P and the glycolytic intermediate fructose-6-phosphate (F6P), whose concentration also rises during the time course. In contrast, the intermediates in the tricarboxylic acid (TCA) cycle showed more complicated changes. The α-ketoglutarate pool consistently expanded by ∼4–16 fold (Figure 1D), but there were no reproducible increases in any of the three other tricarboxylic acid (TCA) cycle components that we measured. Rather, malate, citrate/isocitrate, and fumarate showed less dramatic, coordinated changes and these changes were distinct for each of the three individual time courses (Figure S2).

Our core set of 48 metabolites includes 16 of the 20 amino acids that are incorporated into proteins. Of the 20 amino acids, 11 cannot be synthesized de novo by animals and are often called essential (we include cysteine and tyrosine in the essential group because their synthesis requires the breakdown of other essential amino acids) [14]. While phenyalanine and to a lesser extent histidine increased during early development, the other 7 measured, essential amino acids did not change in abundance. The 9 amino acids that can be synthesized de novo in animal cells are closely derived from glyolytic and TCA intermediates, some via a single transamination step. Several of these amino acids did show strong, consistent changes with aspartate falling ∼3–8 fold and glutamine rising ∼4–7 fold by the late blastula stage (Figure 1E).

To confirm and expand on the metabolomic results, we used amino acid analysis (AAA) to measure the abundance of free amino acids during some early and later stages of development. With one exception (phenylalanine), AAA confirmed the patterns observed in our metabolomic analyses and also provided the absolute abundance of each of the 17 measured amino acid pools (Figure S3). During the early stages of development, polysome content and protein synthesis rates are low but not insubstantial. Amino acid incorporation into proteins has been estimated to increase from ∼50 pmol hr^−1^ amino acid^−1^ embryo^−1^ just following fertilization to ∼150 pmol hr^−1^ amino acid^−1^ embryo^−1^ in late blastulae [15],[16]. Based on our AAA, these low rates would still be sufficient to drain the pools of most essential amino acids over the 11 hr time course. As most of the 9 essential amino acid pools that we could measure remained constant, it is likely that the net rates of protein degradation and synthesis are fairly evenly matched during these early stages of *X.laevis* development.

### Frogs lay eggs with distinct metabolite concentrations and display distinct metabolic changes in response to fertilization

A minority of the metabolite changes we observed in early development were clearly consistent in the single unfertilized eggs and embryos derived from the same cross but were not reproduced in clutches derived from different crosses. This inter-cross variability was particularly evident for the TCA cycle components (Figure S2), as well as for alanine and proline, which showed strong declines in abundance in only one of the three clutches (Figure 1A). By AAA, eggs derived from three different frogs contained three different alanine concentrations (range: ∼0.4–2 mM). But, for all three frogs, the alanine concentration in the resulting embryos rapidly decreased after fertilization and converged to a low level (∼0.1 mM) in late blastulae (Figure S3). It appears that for some metabolites, like proline and alanine, different frogs lay eggs with different quantities of that metabolite. The *X.laevis* frogs used in this study are outbred and so approximate the level of variation found in wild populations. We conclude that in addition to being able to buffer the genetic variation within a population, the developmental program is also robust to some metabolic variation in the egg.

### Alanine is an energy source for early *X.laevis* embryos

It has been proposed that prior to gastrulation, amino acids are the primary source of energy for *X.laevis* embryos [17]. The cognate amino acid(s) was never identified, although very early work had suggested that aspartate levels fall prior to gastrulation in several amphibian species [18]. Here, we have definitively identified alanine and aspartate as amino acids that are consumed during the early development of *X.laevis* (Figure 1A, 1E, Figure S1, S3).

**Figure 2:**
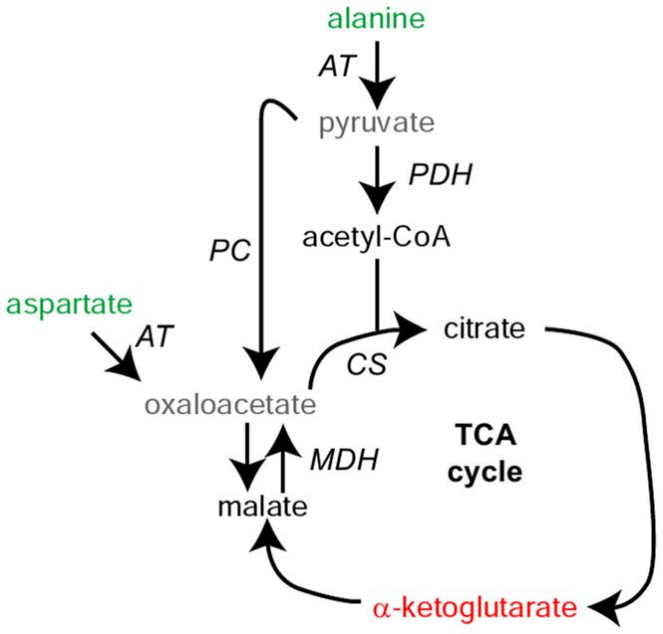
Metabolic fluxes that were traced in early embryos. Metabolites whose abundance increases in early development are shown in red, those whose abundance decreases are in green, those with no observable change are in black, and metabolites that were not measured are in grey. Enzymes are in italics. AT: aminotransferase, PDH: pyruvate dehydrogenase, PC: pyruvate carboxylase, CS: citrate synthase, MDH: malate dehydrogenase.

**Figure 3:**
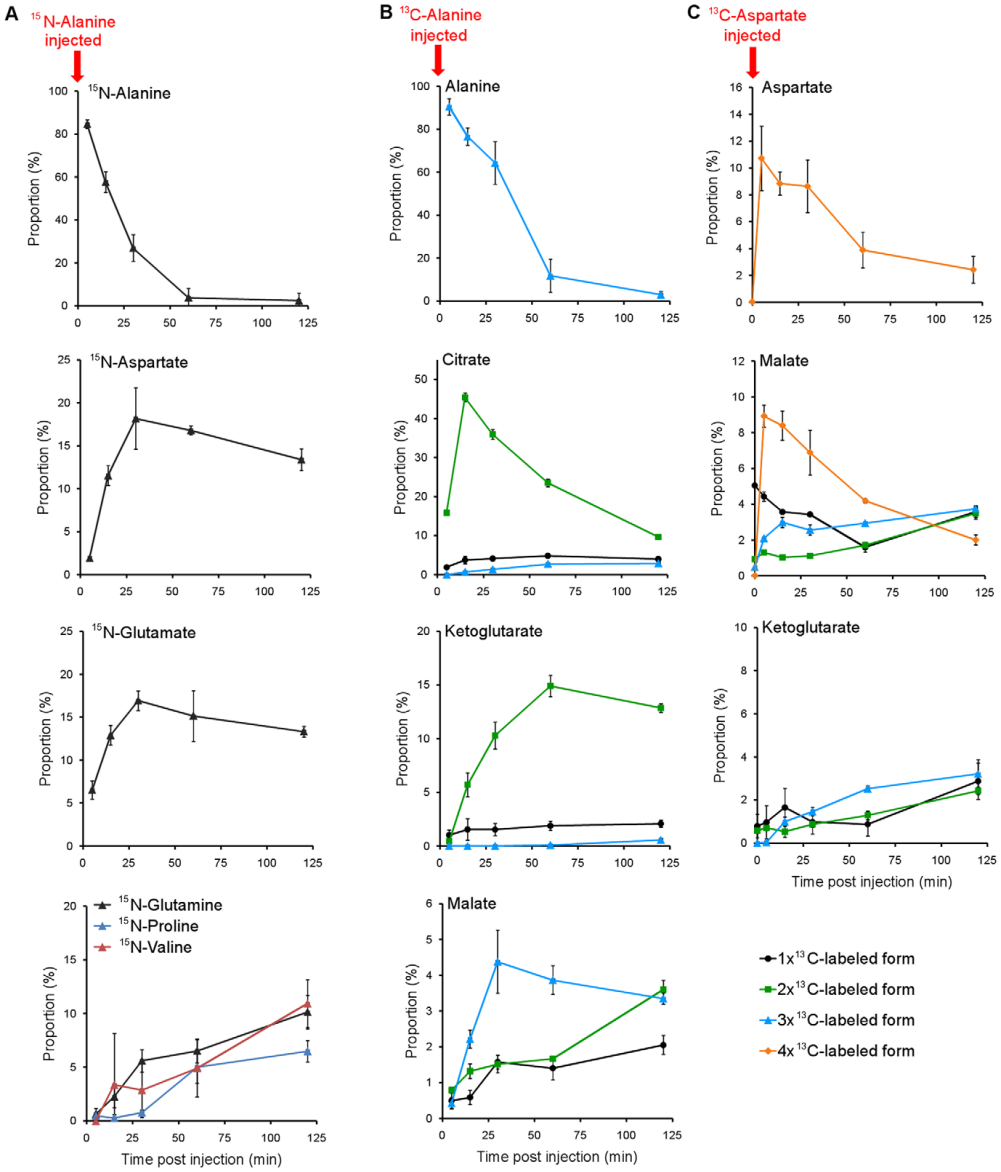
Alanine consumption generates energy in early embryos. (**A**) ^15^N-alanine injected into early embryos is quickly consumed with the heavy nitrogen being transferred to glutamate and aspartate. Glutamine, valine and proline accumulate ^15^N later in the time course. 1 nmol of ^15^N-alanine was injected into 2-cell embryos 2 hours post-fertilization (pf). For the first time point, embryos were quenched as quickly as possible following the injection (∼3 min). The proportion of each of the measurable isotopic forms, relative to the total abundance of that metabolite, was calculated for each embryo at each time point. The isotopic proportions in the 3–4 individual embryos measured at each time point were averaged and plotted. Error bars are ± 1SD. The abundance of each isotopic form was corrected to account for the natural abundance of ^13^C. (**B**) The heavy carbons from U-^13^C-alanine injected into early embryos are transferred to TCA cycle intermediates. Injections and analysis are as for (A). (**C**) U-^13^C-aspartate injected into early embryos almost immediately equilibrates with the malate pool and then moves into the rest of the TCA cycle. Injections and analysis are as for (A), except that 0.5 nmol of U-^13^C-aspartate was injected. Unlike (A) and (B), the first time point (t = 0) is for uninjected 2-cell embryos, and the second time point (t = 3 min) is for embryos that were quenched as quickly as possible following injection.

To better understand the rapid decline in alanine that accompanies fertilization, we injected alanine containing stable, heavy isotopes of nitrogen (^15^N) or of carbon (U-^13^C) into 2-cell embryos and tracked the movement of the isotopes into other metabolite pools by mass spectrometry (Figure 2). The heavy nitrogen from the injected alanine was rapidly transferred into the glutamate and aspartate pools (Figure 3A). The more rapid initial increase in the proportion of labeled glutamate indicates that the dominant enzymatic activity is an alanine-glutamate aminotransferase, with amino groups then being transferred to aspartate by an aspartate-glutamate aminotransferase. The activity of such aminotransferases was further evidenced by reproducible drops in the abundance of α-ketoglutarate, the α-keto acid equivalent of glutamate, and malate, which is likely in equilibrium with oxaloacetate, the α-keto acid form of aspartate (Figure S4). Nitrogen from alanine fluxes through the aspartate and glutamate pools to other amino acids, including valine, proline, and glutamine (Figure 3A). Although we could not directly measure pyruvate, the alanine-glutamate aminotransferase reaction described above will lead to the formation of pyruvate, the α-keto acid equivalent of alanine. Labeled pyruvate should therefore form after injecting alanine with three heavy carbon atoms (U-^13^C-alanine). Consistently, we observed groups of 2 or 3 heavy carbons entering into the pools of TCA cycle intermediates upon injection of U-^13^C-alanine (Figure 3B). Malate with 3 heavy atoms appeared, demonstrating the activity of pyruvate carboxylase (PC), a mitochondrial enzyme that catalyzes the reaction of a bicarbonate ion (which will be predominantly ^12^C) with pyruvate to form oxaloacetate (Figure 3B). Oxaloacetate is expected to be in equilibrium with malate through the action of malate dehydrogenase. PC activity may help restore the pools of TCA intermediates that were partially drained after the alanine injection (Figure S4). In contrast, the rapid accumulation of citrate and α-ketoglutarate with 2 heavy carbons demonstrates the sequential action of pyruvate dehydrogenase and citrate synthase, which catalyze the transfer of 2-carbon units from pyruvate into the TCA cycle for the purpose of generating ATP.

We used the same approach to track the fate of the aspartate consumed during early development. Nearly immediately upon being injected (<3 min) into the 2-cell embryo, the heavy carbon chain of U-^13^C-aspartate containing four ^13^C atoms was equilibrated with malate, suggesting a rapid flux through aspartate-glutamate aminotransferase and malate dehydrogenase (Figure 3C). The heavy carbon chain was then distributed around the TCA cycle as seen in the rise of α-ketoglutarate labeled with three ^13^C atoms.

The metabolic fluxes that we observed in early embryos are summarized in Figure 2. Based on our data as a whole, we propose that the decline in aspartate and the rise in glutamine are linked. By AAA, embryos consume ∼2 nmol of aspartate by the late blastula stage but produce ∼1 nmol of glutamine (Figure S3). By this stoichiometry, the nitrogen is roughly accounted for and free ammonium levels would not rise, which is known to be the case [19]. In this view, the egg is stockpiled with aspartate, which acts as a source of nitrogen for generating glutamine for the embryo. Although we are unsure what the benefit of these changes to the embryo are, as aspartate and glutamine are the two nitrogen providers for nucleotide biosynthesis, this flux may help ensure adequate availability of usable nitrogen for nucleotide biosynthesis.

### Declining dATP pool: reconsidering an old model for the MBT

**Figure 4:**
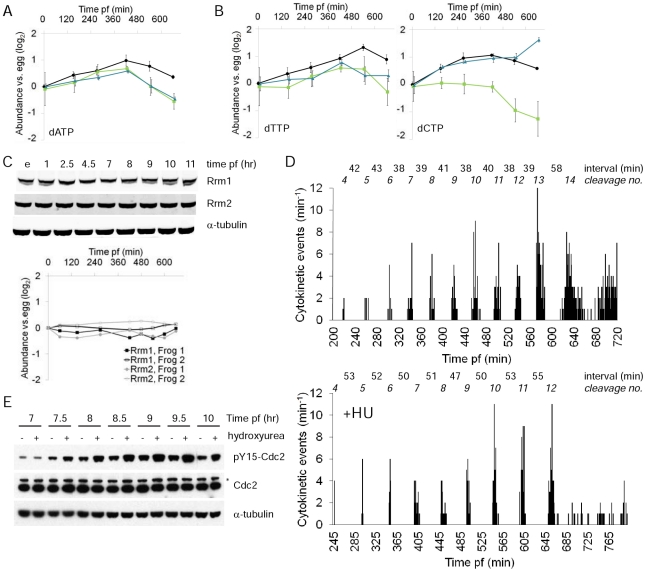
Evidence suggesting that dATP limitation triggers the MBT. (**A,B**) Plots of dATP, dTTP, and dCTP abundance during early development. dGTP was not measurable in part due to interference from ATP, which is much more abundant. The MBT occurs 8 hours post-fertilization (pf), see Figure 1B. Results plotted as for Figure 1C. Error bars are ± 1SD. (**C**) The two subunits of ribonucleotide reductase (Rrm1, Rrm2) do not change in abundance during early development. Western blotting of unfertilized eggs and embryos from two different females frogs was quantified (bottom panel). (**D**) Time lapse imaging of the MBT in unperturbed embryos (top panel) and embryos incubated in 20 mM hydroxyurea (HU, bottom panel). The animal pole of single embryos was imaged and the timing of each cytokinesis recorded. The time interval between each synchronous group of cell divisions is noted. The two embryos are descended from different frogs on different days. There was notable variation in the time interval between cleavage events in otherwise unperturbed, imaged embryos (data not shown), so the longer intervals in HU treated embryo are not necessarily due to HU toxicity. (**E**) Hydroxyurea causes hyperphosphorylation of Y15 on Cdc2 beginning at the MBT, 7.5 hours after fertilization. Embryos were incubated at 18°C for 3 hours and then the temperature slowly raised to 23°C, accounting for the slightly more rapid entry into the MBT than in Figure 1B. * indicates a non-specific band.

We observed reproducible changes in the abundance of dATP (Figure 4A). dATP levels rose following fertilization and then started falling around the time of the MBT (∼8 hrs post-fertilization). There was a ∼2-fold change between the peak concentration observed at 7 hours after fertilization and the concentration measured in late blastulae, at 11 hours post fertilization. dTTP and dCTP pools exhibited more variable, clutch-dependent patterns and dGTP could not be detected by our methods (Figure 4B). While not as dramatic as many of the changes we observed, these changes were nevertheless somewhat surprising, as it is thought that cells tightly control dNTP concentrations [20]. All four dNTPs are synthesized through the action of ribonucleotide reductase (RNR). A complex set of allosteric feedback loops are thought to allow the four dNTP pools to achieve the desired concentrations and stoichiometry, in large part by controlling the activity of RNR towards each of its substrates. But the dNTPs clearly did not all rise and fall in perfect tandem in our data and it has been previously reported that dNTP pool sizes can change differentially. For instance, in mammalian cells, dATP pools are far more sensitive to inhibition of RNR by the small molecule inhibitor hydroxyurea (HU) than are the other dNTP pools [21], [22].

We considered potential causes of the decline in dATP around the time of the MBT. We observed no change in the abundance of either ribonucleotide reductase (RNR) subunit (Figure 4C). It also seemed unlikely that the declines in dATP abundance were caused by changing cell cycle distributions in the ∼4000–8000 cell embryo at the MBT, since S-phase is when dNTP levels would be expected to be highest if these levels were cell cycle regulated at later stages of development [23], , but the first cell cycle phase to be extended at the *X.laevis* MBT is the S-phase [27]. In fact, the proportion of each cell cycle devoted to S-phase does not decrease as cells traverse the MBT and cell cycles elongate in late blastulae [27].

We reasoned that the embryo’s dATP pool might decline around the MBT due to the increasing demands of DNA synthesis. The number of genomes increases exponentially during the cleavage cycles but the length of S-phase remains constant (∼20 min) until the MBT [27]. Therefore, for the embryo as a whole, the rate of dNTP fixation into DNA also increases exponentially. We used time lapse imaging of the animal poles of intact embryos to re-examine the timing of the MBT. We found that cells at the animal pole of *X.laevis* progressed through 13 synchronous cleavage cycles but the 13^th^ cycle was sometimes, but not always, noticeably longer (Figure 4D). When the 13^th^ cycle was longer, it was a <20% increase (n = 5). The 13^th^ cycle is one cycle later than was reported in two studies of cells from dissociated embryos [7], [8], but is consistent with a previous report in which intact embryos were imaged [6], [28]. Also as previously reported, incubating embryos with HU led to few subsequent divisions after the 12^th^ cleavage cycle, and embryo death (Figure 4D) [28]. As HU will prevent the synthesis of additional dNTPs by RNR, this result strongly suggests that embryonic pools of at least one dNTP are functionally exhausted around the time of the MBT, leading to cell cycle arrest in S-phase and cell death. As the embryo contains ∼4096 cells after 12 synchronous divisions, these experimental findings are fairly consistent with the finding that unfertilized *X.laevis* eggs contain ∼10 pmol of each dNTP which would suffice to synthesize ∼2500 *X.laevis* genomes [29].

We consistently observed, however, that imaged embryos went through the cleavage cycles more slowly than non-imaged siblings (Figure S5). This phototoxicity was present even when light exposures/intensities were kept to the minimum necessary to obtain reasonable images (3s of light every minute) and when all UV wavelengths were filtered out. Despite their slower cleavage cycles, imaged embryos did go on to develop normally (data not shown), perhaps explaining why this phototoxicity had not been previously reported. We were concerned about the effects this mild phototoxicity could have on the relative timing of events. To confirm the coincidence between the timing of the MBT and the time at which cell cycles arrest in response to HU treatment, we measured the abundance of pY15-Cdc2 in embryos that were only exposed to normal room lighting in the presence or absence of HU (Figure 4E). This alternative approach demonstrated that HU does not affect the embryonic cell cycle until the MBT, confirming the functional exhaustion of dNTP pools at the MBT.

The coincidence between the time at which maternal pools of dNTPs are exhausted and the MBT, as well as our novel result that dATP pools are diminished around the time of the MBT, support an old idea that the MBT is triggered by declining dNTP pools [7],[30]. In this model, the MBT occurs when dNTP pools and the biosynthetic capacity of RNR are outpaced by the exponentially increasing demands for dNTPs at replication forks. Although dATP does not decrease dramatically in abundance (∼2-fold, Figure 4A), replication forks in yeast arrest when dNTP concentrations are only 20% lower than normal [31]. One significant problem with the limiting dNTP model is how difficult it has been to definitively test. Newport and Dasso were unable to repeat the results of Lovtrup and colleagues, who claimed that injecting dNTPs could increase the number of sychronous cleavage cycles in *X.laevis* [28], [30]. When we attempted this experiment, we consistently found that injecting dNTPs to 100–300 µM, or ∼2.5–7.5 fold their endogenous concentration, slowed the cleavage cycles relative to control siblings. The unknown toxic effects of dNTP injection made further interpretation treacherous.

To our knowledge, the limiting dNTP model is consistent with every fact that is known about the MBT in *X.laevis*. The MBT occurs when the DNA:cytoplasm ratio increases above a critical threshold [7], [32]. In the limiting dNTP model, the amount of DNA is represented by the number of replication forks that are fixing dNTPs into polymer, while the cytoplasm is represented by dNTP pools and RNR enzyme. As frog eggs have been shown to quickly form nuclei around injected plasmids [33], and frog extracts as well as frog eggs replicate injected plasmid DNA [34], [35], the limiting dNTP model is compatible with experiments showing that injected plasmids can cause a precocious MBT [36]. It has been demonstrated that the ATR-Chk1 replication checkpoint is transiently activated during the MBT [13]. In the limiting dNTP model, depleted dNTP pools would cause replication forks to stall after ∼12–13 replication cycles, activating the ATR-Chk1 checkpoint pathway to retard origin firing and extending S-phase. ATR-Chk1 is known to be the primary signaling route for stalled replication forks in animal cells and, in *X.laevis* egg extracts, ATR signaling has been shown to suppress origin firing [37], [38].

As the orthologs of Chk1 and ATR are required to slow the rapid replication cycles of syncytial *Drosophila* embryos and to coordinate cell size and cell cycle time in early *C.elegans* embryos [39], [40], limiting dNTPs could be a conserved feature of early animal development, possibly including mammals [5]. An attractive feature of the limiting dNTP model is that it provides a mechanistic explanation for how the MBT occurs so reproducibly from embryo-to-embryo. That is, rather than requiring the female frog to accurately load each egg with a certain amount of a limiting protein, embryo-to-embryo variation in dNTP pool sizes would be intrinsically low due to allosteric control over RNR.

It is intriguing what this model, if true, would say about relations between cell size and the cell cycle. The average embryonic frog cell after 13 cleavage divisions has ∼60 pL of non-yolk volume and is much too large to be affected by the cell size requirement for the G1/S transition that has been surmised to exist in typical somatic cells (e.g. a HeLa cell is ∼2 pL) [41], [42]. The limiting dNTP model suggests that diminishing cell size first limits the speed of the cell cycle by decreasing the absolute rate of the metabolic fluxes required for DNA synthesis. Perhaps this would not be so surprising. In most cells, dNTPs need to be synthesized in real-time during DNA replication. Even for the largest dNTP pool in mouse fibroblasts (dTTP) [22], the amount of dTTP that needs to be polymerized to duplicate the genome is greater than 40 times the pool size.

## Materials and Methods

### Molecular biology, chemicals, antibodies

Standard molecular biology techniques were used [43]. Unless otherwise specified, chemicals and enzymes were from Sigma-Aldrich (St. Louis, MO, U.S.A.). Antibodies used were: α-Cdc2 (sc-54, Santa Cruz Biotechnology, Santa Cruz, CA, U.S.A.), α-pY15-Cdc2 (#9111, Cell Signaling Technology, Beverly, MA, U.S.A.), α-alpha-tubulin (MS-581, Lab Vision/Thermo-Scientific, Fremont, CA, U.S.A.), α-Rrm1 (10526-1-AP, ProteinTech Group Inc., Chicago, IL, U.S.A.), and α-Rrrm2 (sc-10846, Santa Cruz Biotechnology).

### Embryo quenching, metabolite extraction, metabolomic analysis

Standard *X. laevis* techniques were typically followed [44]. Embryos were washed 4 times in dH_2_0 and transferred individually to an eppendorf tube. For each tube, as much water as possible was removed with a pipette and the embryo was quenched by addition of 55 µL of cold (−20°C) 2∶2∶1 acetonitrile:methanol:dH_2_0 (all solvents were HPLC grade) and immediate vortexing. Vortexing in this solvent mixture rapidly (<5s) lysed and dispersed the embryo. All remaining steps were carried out at 4°C. After 20 minutes on ice, precipitates (proteins, DNA, RNA, etc.) were pelleted by centrifugation (20,000 g, 5 min) and the supernatant isolated. The pellet was extracted with 25 uL of the solvent mixture and the above steps repeated to obtain ∼80 uL of metabolite extract. Metabolites were shipped overnight at −20°C from Boston, MA to Princeton, NJ for LC-MS/MS metabolomic analysis the following day. Metabolites were quantified as described [45].

### Amino acid injections

Dejellied, two-cell embryos derived from in vitro fertilization of a single female/male pair were transferred into 0.1xMMR 5% Ficoll. The marginal zone of each cell was injected with either 10 nL of 50 mM ^15^N-L-alanine, 50 mM U-^13^C-L-alanine, or 25 mM U-^13^C-L-aspartate (all from Cambridge Isotope Laboratories, Andover, MA, U.S.A.). Injection solutions were adjusted to pH 7.7–7.9, the measured pH of *X.laevis* early embryo cytoplasm [46], and filter sterilized. Embryos were injected for ∼3 min to generate a cohort of embryos that were individually quenched as above at a given time point. Multiple ∼3 min injected cohorts were generated so there were sufficient cohorts for all the time points. For the zero time point (alanine injections) or the 3 min time point (aspartate injection), four embryos were quickly injected, then all four were individually quenched as above. This process took ∼3 min from the first injection to the last quenching. For each metabolite, the abundance of the unlabeled form, as well as the labeled forms, were corrected for the natural abundance of ^13^C (1.1%).

### Alternative method for metabolite extraction from embryos for AAA and for validation of metabolomics data

For AAA, three clutches of embryos were generated by in vitro fertilization of eggs from 3 individual females. At each time point, for each independent clutch, 30 embryos were washed extensively in distilled water, transferred to an eppendorf tube, and all but ∼100–200 µL of water removed prior to flash-freezing in liquid nitrogen. Frozen embryos were stored at −80°C. To prepare deproteinated extracts, 1 mL of ice-cold 2% sulfosalicylic acid was added to frozen embryos and the embryos were fully lysed and dispersed by sonication for 20s. This mixture was kept on ice for 30 minutes and the precipitate pelleted by centrifugation (5 min, 20,000 g, 4°C). 135–140 µL of each deproteinated extract was directly subjected to AAA without prior to hydrolysis in hydrochloric acid at the Molecular Biology Core Facility of the Dana Farber Cancer Institute, Boston, MA, USA.

For metabolomic analysis by the Mootha Laboratory (Broad Institute of MIT and Harvard, Cambridge, MA, USA), three clutches of embryos were generated by in vitro fertilization of eggs from three individual females. At each time point, for each independent clutch, 20 embryos were washed extensively in distilled water transferred to an impact-resistant tube, and all but ∼25–50 µL of water removed prior to flash-freezing in liquid nitrogen. Frozen embryos were stored at −80°C. To prepare deproteinated extracts, 100 µL of ice-cold glass beads were added on top of the frozen embryos and cold (−20°C) 85% ethanol added to obtain a 500 µL total volume. Embryos were lysed and dispersed by bead beating (30s, 4°C, Fastprep FP120A, MP Biomedical, Solon, OH, U.S.A) and precipitates pelleted by centrifugation (15 min, 20,000 g, 4°C). 200 µL of each supernatant was then dried under liquid nitrogen and subjected to metabolomic analysis as described [47].

### Time-lapse microscopy

The animal pole of early *X.laevis* embryos was imaged with a CCD-camera (Micropublisher 5.0 cooled RTV, Qimaging, Surrey, BC, Canada) connected to a dissecting scope (SMZ1500, Nikon, Melville, NY,U.S.A.) at 40x magnification. A single, dejellied 16-64-cell embryo was placed on top of Nitex screen in a dish of 0.1xMMR with 50 µg/mL gentamicin at 18°C. The light source was flexible fiber-optic light guides transmitting light from a halogen lamp with an infrared interference filter (Schott-Fostec, Elmsford, NY, U.S.A.). The light from each guide was passed through a green filter (bandpass: ∼400–650 nm, VG-9, Edmund Optics, Barrington, NJ, U.S.A.). Despite light filtering and the least intense and shortest possible light exposures consistent with obtaining a high quality image (3 s/min of imaging), phototoxicity was evident. Sibling embryos that were not subjected to light aside from normal room lighting were reared in the same medium and on the same microscope stage to ensure the same temperature as the imaged embryo. Throughout the ∼10 hr time course, the imaged embryo always had drastically fewer cells than control siblings, demonstrating the slowing of the cleavage cycles by light exposure. But the difference between the imaged embryo and the control siblings was variable from one batch of eggs to the next, suggesting that sensitivity to phototoxicity was variable. Despite the phototoxicity, imaged embryos went on to develop normally. Time-lapse movies were analyzed for cell division events and the time at which each cleavage plane first appeared was recorded.

## Supporting Information

Figure S1 Remodeling of the metabolome during early development was confirmed with a separate metabolomic system. Three time points in early development were analyzed: unfertilized eggs (t = 0), stage 6 embryos (t = 6 hr pf), and stage 9 embryos (t = 10 hr pf). Eggs/embryos were derived from three different female frogs (frog 1: black circles, frog 2: red squares, frog 3: blue diamonds). The y-axes indicate metabolite abundance per egg or embryo in arbitrary units (AU). Metabolites were measured with a distinct LC-MS/MS system in the Mootha Laboratory and with different quenching and extraction techniques (see Materials and Methods). Despite these experimental differences, the overwhelming majority of metabolites measured by both systems showed a consistent pattern. The 25 metabolites presented are those most relevant to the results presented in the main text.
(TIF)

Figure S2 The three TCA cycle components in the core 48 metabolites showed patterns that, while similar between the three components, were distinct to each clutch of eggs/embryos. As in Figure 1C, for each single egg or embryo, each metabolite abundance was divided by the average egg value for that metabolite. This ratio was then log_2_ transformed. At each of six time points, the 3-4 individual log_2_ ratios was averaged and plotted, with error bars indicating 1SD in either direction. The three clutches were plotted separately (black: clutch 1, green: clutch 2, blue: clutch 3).
(TIF)

Figure S3 Amino acid analysis determined free amino acid concentrations in embryos during early development (0–10 hrs, comparable to metabolomic analyses) and in later development (>10 hrs, no comparable data in metabolomic analyses). Extracts were prepared with a different technique than was used in either metabolomic analysis (see Materials and Methods). Eggs/embryos were derived from three different female frogs (frog 1: black circles, frog 2: red squares, frog 3: blue diamonds). Cys, ser, and trp could not be quantitated. Total amino acids (bottom right panel) is the sum of the 17 measured amino acids.
(TIF)

Figure S4 Following injection of either ^15^N-alanine or U-^13^C-alanine at t = 0, a temporary drop in the pool sizes (total abundance) of α-ketoglutarate and malate was observed. Graphs display total abundance of individual metabolites expressed in arbitrary units (AU). Total abundance is the sum of all the measurable isotopic forms and the unlabeled form of that metabolite.
(TIF)

Figure S5 Embryos subjected to time-lapse imaging showed noticeable phototoxicity, even when light intensities and exposure times were minimized. An imaged embryo (center) and six sibling control embryos (two rows of three embryos) are shown. The imaged embryo has larger cells due to slower cleavage cycles. Control embryos were reared in the same dish on the microscope stage but were not exposed to light from the microscope’s lamp (room lights were on).
(TIF)

Table S1 Metabolite levels expressed relative to the average value measured for that metabolite in the egg samples. On three different days, three different male/female pairs were bred in vitro to obtain clutches of unfertilized eggs (time = 0) and early embryos at various times post-fertilization (pf) at18°C. Single eggs or embryos were rapidly quenched, extracted, and subjected to metabolomic analysis. The ratios were calculated separately for the three different clutches. For each time point average and standard deviation values of n = 3–4 are reported.
(XLS)
