# Supplementary figures and images for: Remodeling of the Metabolome during Early Frog Development
Source: PLoS One. 2011 Feb 4;6(2):e16881. doi: 10.1371/journal.pone.0016881 (PMC3035664; doi:10.1371/journal.pone.0016881)

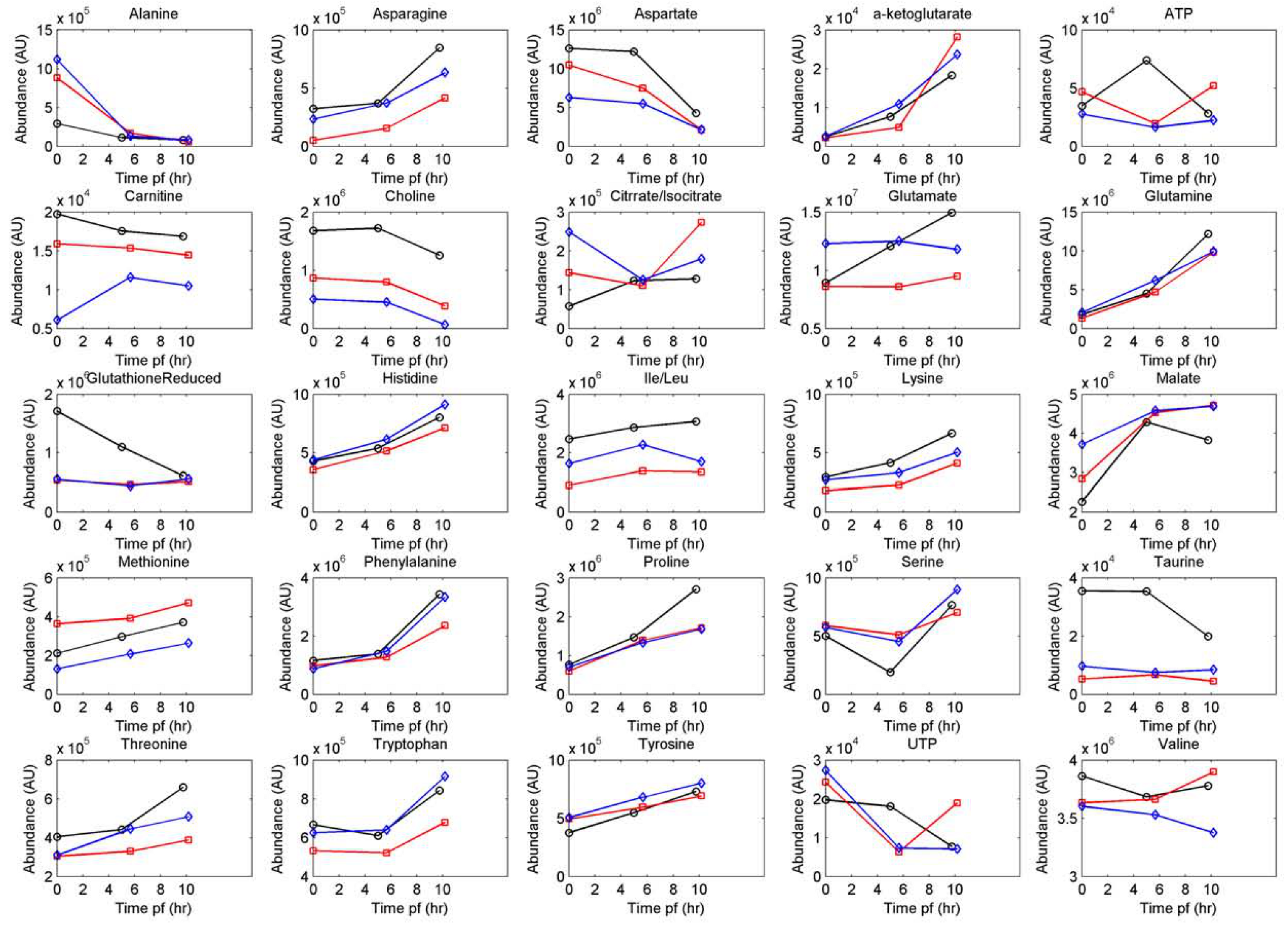

Supplement: Figure S1 — Remodeling of the metabolome during early development was confirmed with a separate metabolomic system. Three time points in early development were analyzed: unfertilized eggs (t = 0), stage 6 embryos (t = 6 hr pf), and stage 9 embryos (t = 10 hr pf). Eggs/embryos were derived from three different female frogs (frog 1: black circles, frog 2: red squares, frog 3: blue diamonds). The y-axes indicate metabolite abundance per egg or embryo in arbitrary units (AU). Metabolites were measured with a distinct LC-MS/MS system in the Mootha Laboratory and with different quenching and extraction techniques (see Materials and Methods). Despite these experimental differences, the overwhelming majority of metabolites measured by both systems showed a consistent pattern. The 25 metabolites presented are those most relevant to the results presented in the main text. (TIF) [file pone.0016881.s001.tif]

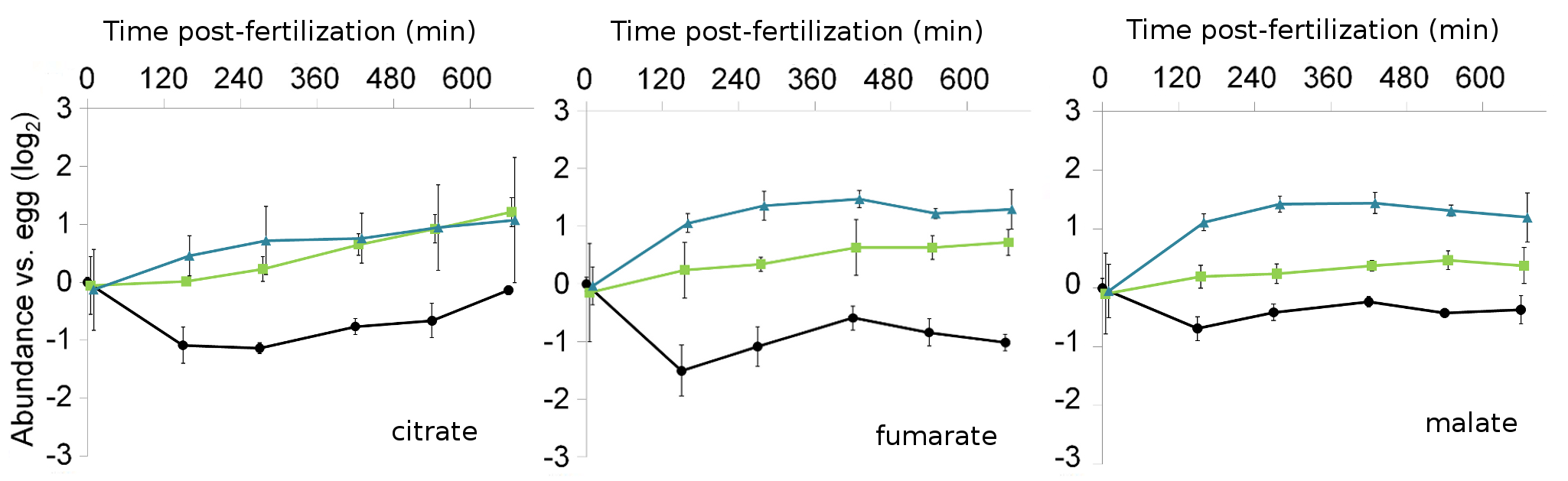

Supplement: Figure S2 — The three TCA cycle components in the core 48 metabolites showed patterns that, while similar between the three components, were distinct to each clutch of eggs/embryos. As in Figure 1C, for each single egg or embryo, each metabolite abundance was divided by the average egg value for that metabolite. This ratio was then log2 transformed. At each of six time points, the 3-4 individual log2 ratios was averaged and plotted, with error bars indicating 1SD in either direction. The three clutches were plotted separately (black: clutch 1, green: clutch 2, blue: clutch 3). (TIF) [file pone.0016881.s002.tif]

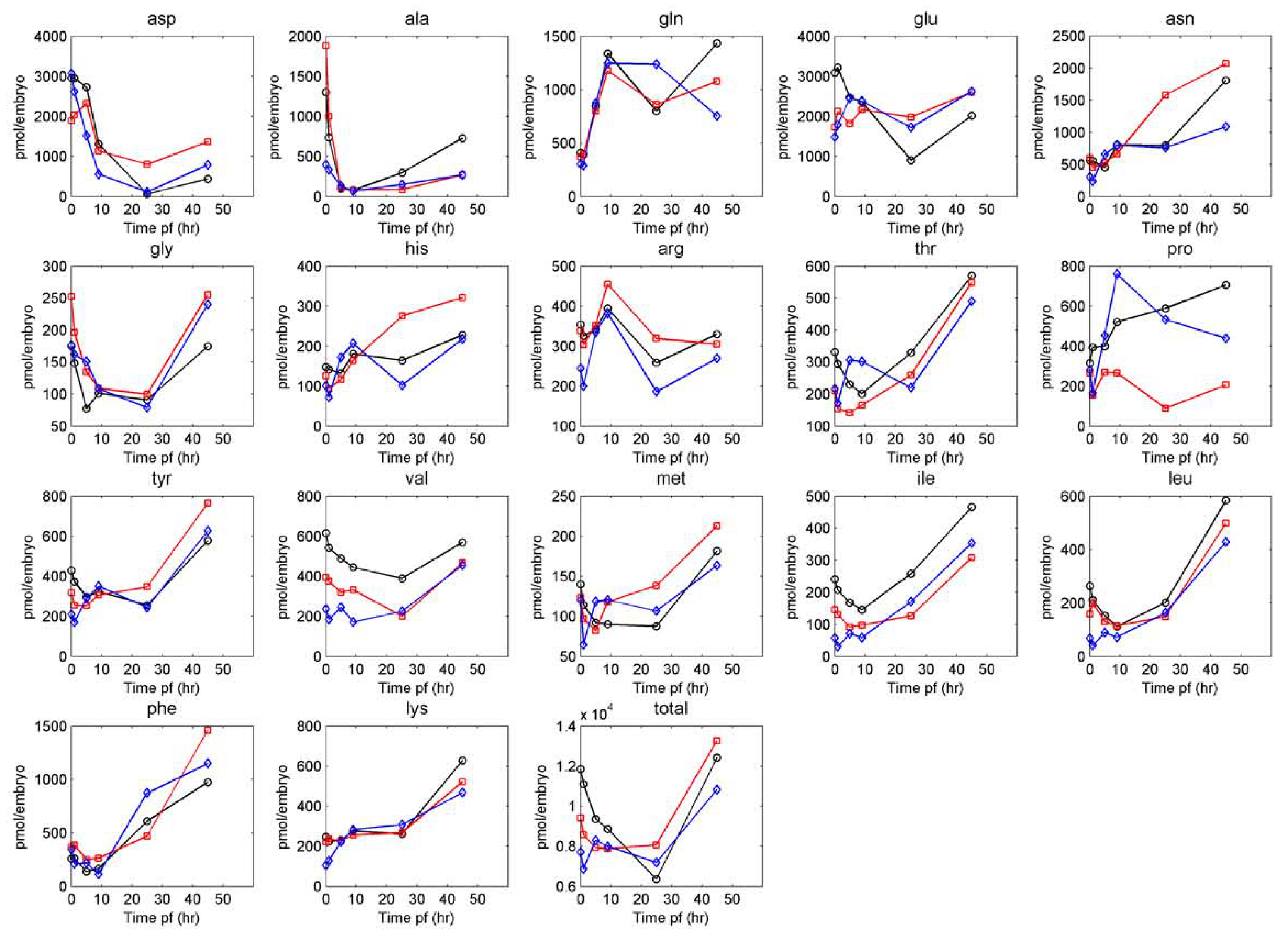

Supplement: Figure S3 — Amino acid analysis determined free amino acid concentrations in embryos during early development (0–10 hrs, comparable to metabolomic analyses) and in later development (>10 hrs, no comparable data in metabolomic analyses). Extracts were prepared with a different technique than was used in either metabolomic analysis (see Materials and Methods). Eggs/embryos were derived from three different female frogs (frog 1: black circles, frog 2: red squares, frog 3: blue diamonds). Cys, ser, and trp could not be quantitated. Total amino acids (bottom right panel) is the sum of the 17 measured amino acids. (TIF) [file pone.0016881.s003.tif]

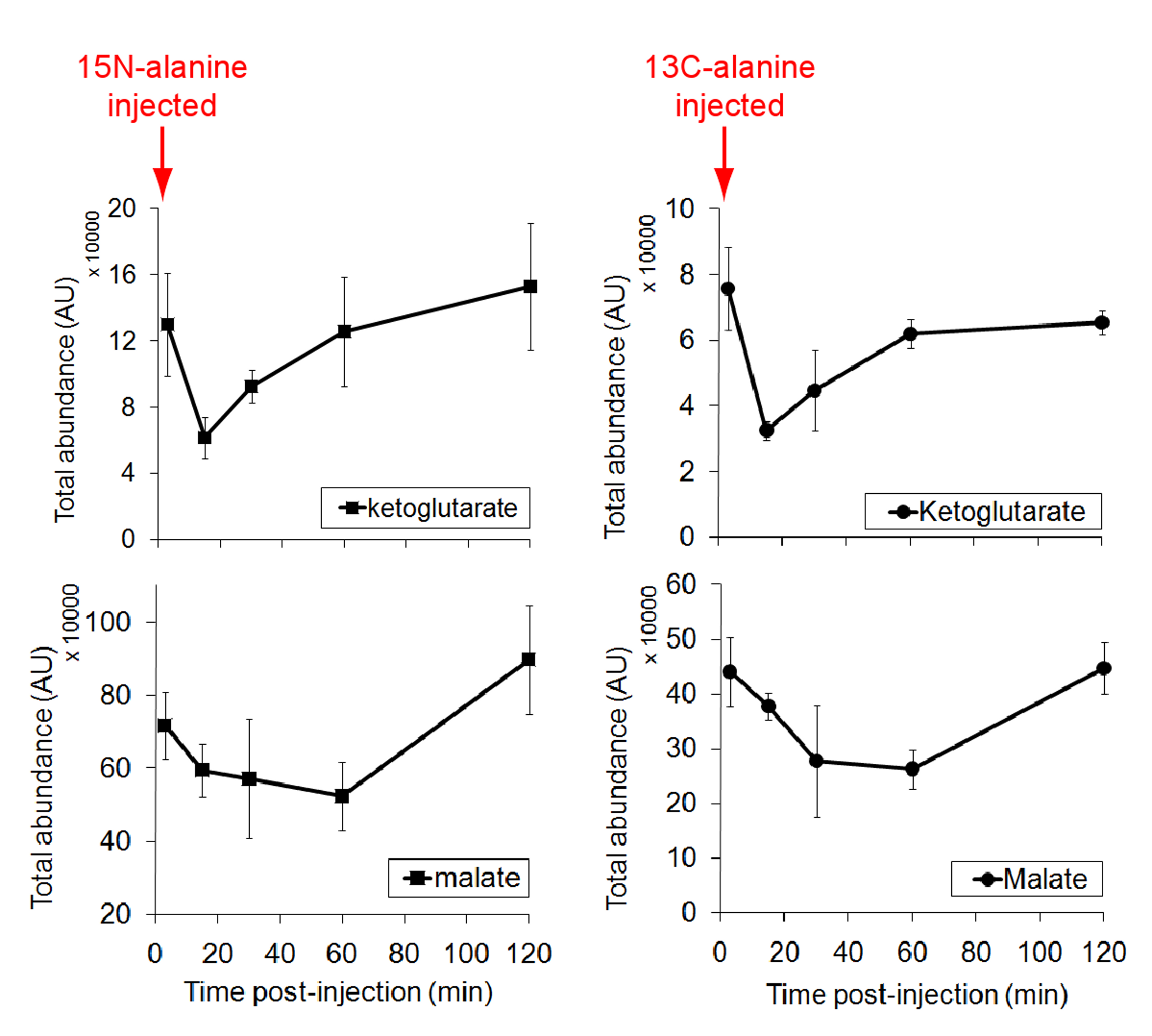

Supplement: Figure S4 — Following injection of either 15N-alanine or U-13C-alanine at t = 0, a temporary drop in the pool sizes (total abundance) of α-ketoglutarate and malate was observed. Graphs display total abundance of individual metabolites expressed in arbitrary units (AU). Total abundance is the sum of all the measurable isotopic forms and the unlabeled form of that metabolite. (TIF) [file pone.0016881.s004.tif]

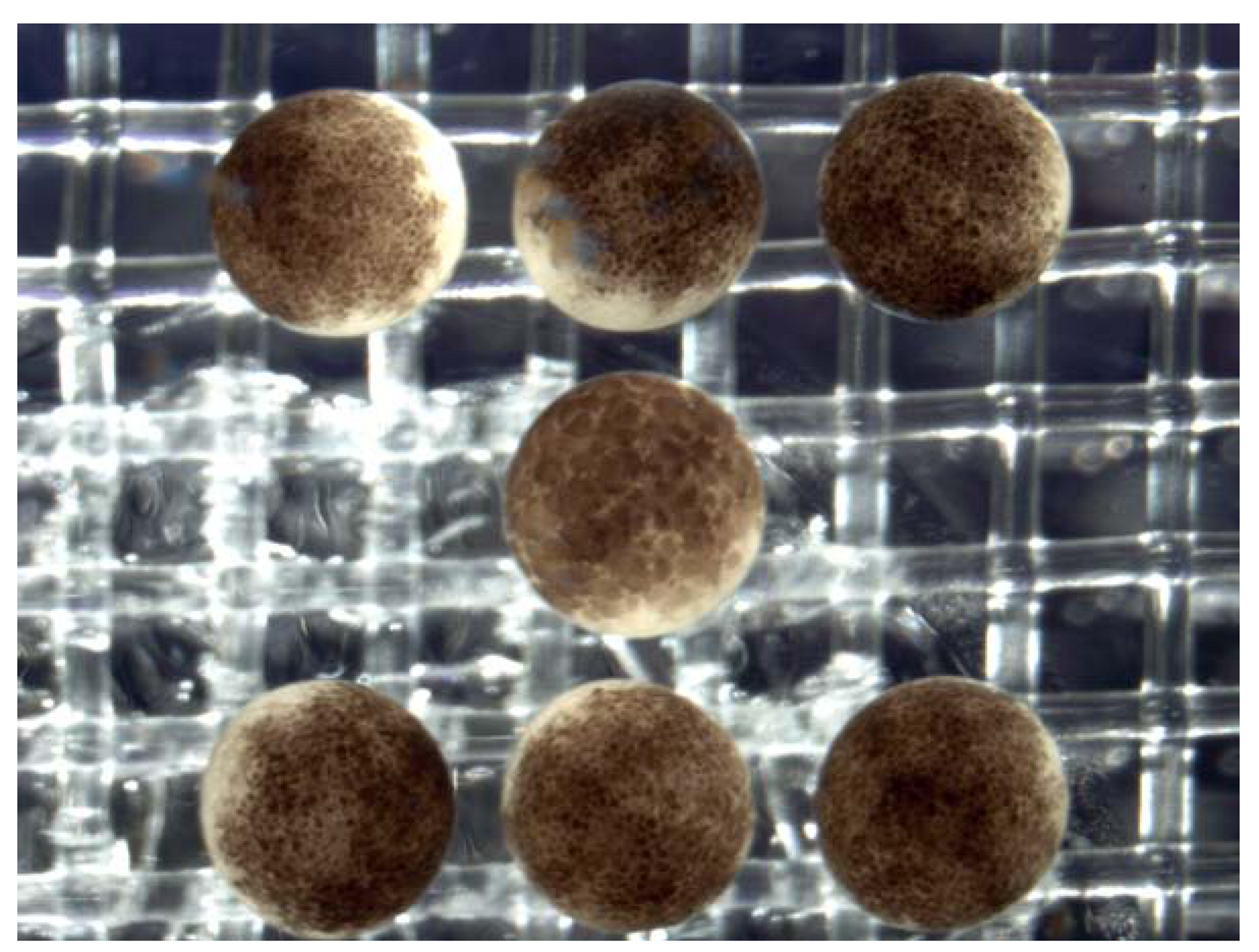

Supplement: Figure S5 — Embryos subjected to time-lapse imaging showed noticeable phototoxicity, even when light intensities and exposure times were minimized. An imaged embryo (center) and six sibling control embryos (two rows of three embryos) are shown. The imaged embryo has larger cells due to slower cleavage cycles. Control embryos were reared in the same dish on the microscope stage but were not exposed to light from the microscope’s lamp (room lights were on). (TIF) [file pone.0016881.s005.tif]
